# Application of Bayesian causal inference and structural equation model to animal breeding

**DOI:** 10.1111/asj.13359

**Published:** 2020-03-23

**Authors:** Keiichi Inoue

**Affiliations:** ^1^ National Livestock Breeding Center Nishigo Fukushima Japan

**Keywords:** animal breeding system, Bayesian causal inference, external intervention, inductive causation algorithm, structural equation model

## Abstract

Optimized breeding goals and management practices for the improvement of target traits requires knowledge regarding any potential functional relationships between them. Fitting a structural equation model (SEM) allows for inferences about the magnitude of causal effects between traits to be made. In recent years, an adaptation of SEM was proposed in the context of quantitative genetics and mixed models. Several studies have since applied the SEM in the context of animal breeding. However, fitting the SEM requires choosing a causal structure with prior biological or temporal knowledge. The inductive causation (IC) algorithm can be used to recover an underlying causal structure from observed associations between traits. The results of the papers, which are introduced in this review, showed that using the IC algorithm to infer a causal structure is a helpful tool for detecting a causal structure without proper prior knowledge or with uncertain relationships between traits. The reports also presented that fitting the SEM could infer the effects of interventions, which are not given by correlations. Hence, information from the SEM provides more insights into and suggestions on breeding strategy than that from a multiple‐trait model, which is the conventional model used for multitrait analysis.

## 1 INTRODUCTION

In animal breeding strategies, there are two types of breeding goal to ensure the ability of animal to fit the demands of producers, consumers, and markets. One is the fundamental or sustainable breeding goal, which is not dependent on market trends and focuses on properties such as reproduction, feed efficiency, and longevity. The other is the flexible breeding goal that reflects temporal market or economical demands and focuses on properties such as the qualities of milk and meat products. When we are confronted with the need to improve a new trait because of changing market demands, such as fatty acid composition, which is a meat quality trait and is related to the sweet aroma (Sakuma et al., 2012) and flavor of beef (Suzuki, Yokota, Shioura, Shimazu, & Iida, 2013) in Japanese Black cattle, we add the new trait to conventional selection traits and improve multiple traits simultaneously. In such a case, the most important thing is to confirm the genetic correlations between the traits by estimating the genetic parameters fitted by the multiple‐trait model (MTM; Henderson & Quass, 1976) to prevent negative effects on the conventional economical traits. However, there are not only ‘correlations’ but there could also be ‘causal relationships’ between the traits. For example, in the case of the relationship between gestation length (GL) and calving difficulty (CD), GL affects CD, because a longer GL causes a bigger infant or harmonization. In contrast, the opposite relationship is not possible; CD never affects GL under biological conditions.

When causal relationships exist between traits, fitting a structural equation model (SEM) allows for inferences of the magnitude of the causal effects between traits (Haavelmo, 1943; Wright, 1921). SEMs have been applied in various fields, such as economics, psychology, and sociology. In recent years, Gianola and Solensen (2004) proposed adaptations of the SEM in the context of quantitative genetics and mixed‐effect models. Ever since their proposal, several studies have applied the SEM in the context of animal breeding in dairy goats (de los Campos, Gianola, Boettcher, & Moroni, 2006), dairy cattle (de los Campos, Gianola, & Heringstad, 2006; Heringstad, Wu, & Gianola, 2009; Konig, Wu, Gianola, Heringstad, & Simianer, 2008; Wu, Heringstad, Chang, de los Campos, & Gianola, 2007; Wu, Heringstad, & Gianola, 2008; etc.), pigs (Ibanez‐Escriche, Lopez de Maturana, Noguera, & Varona, 2010; Varona, Sorensen, & Thompson, 2007), and beef cattle (Inoue, Hosono, & Tanimoto, 2017; Inoue et al., 2016).

Fitting the SEM requires choosing a causal structure with prior biological or temporal knowledge. However, the inductive causation (IC) algorithm (Pearl, 2000; Verma & Pearl, 1990), which is one of the Bayesian causal inference, can be used to recover an underlying causal structure from observed associations between traits. The IC algorithm can output the causal relationships with a directed acyclic graph (DAG), which is a set of variables connected by directed edges, or a partially oriented graph. Searching for the causal structure in the algorithm is based on a conditional independence (Dawid, 1980) between variables.

Valente, Rosa, de los Campos, Gianola, and Silva (2010) adapted the IC algorithm to a mixed‐model context and showed that applying this method to the posterior distribution of the residual (co)variance matrix of a standard MTM recovered the expected network in simulated data. They also presented an application of the methodology to real field data of European quail. After their work, several groups have tried to explore the causal structure from the real field data.

This review will first describe an overview of the IC algorithm and SEM, and then introduce some papers that apply these techniques to the context of animal breeding.

## 2 THE IC ALGORITHM

**FIGURE 1:**
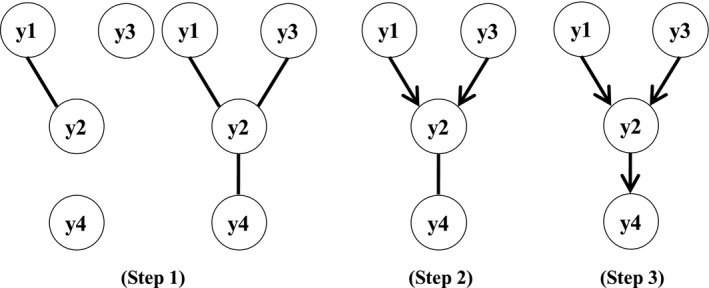
An example of the steps for the inductive causation algorithm. y1 to y4 represent traits, ‘‐’ and ‘→ (←)’ represent undirected and directed edges, respectively

The IC algorithm, which was proposed by Pearl (2000) and Verma and Pearl (1990), allows to search for causal networks. The IC algorithm performs a series of statistical decisions based on partial correlations between traits and consists of the following three steps (an example is shown in Figure 1):

### 2.1 Step 1

If all partial correlations of two traits conditional on each possible set of the other traits are different from 0, the two traits are connected by an undirected edge (e.g., y1 − y2). For example, when there are four target traits for inferring the causal structure (y1, y2, y3, and y4), as shown on the left graph of Step 1 in Figure 1, if all partial correlations of two arbitrary traits (e.g., y1 and y2) conditional on every set of the other traits (i.e., ρ_y1y2|y3_, ρ_y1y2|y4_, and ρ_y1y2|y3y4_) and correlation between y1 and y2 (ρ_y1y2_) are different from 0 (significant), the two traits are connected by an undirected edge (y1 − y2). The graph with undirected edges is finally generated by iterating the same process for the remaining possible set of traits (the right graph of Step 1 in Figure 1).

### 2.2 Step 2

In the undirected graph obtained by step 1, if partial correlations of two nonadjacent traits (e.g., y1 and y3) with a common adjacent trait (e.g., y2 in y1 − y2 − y3) are dependent conditional on any possible set including the adjacent trait (y2), that is, all partial correlations (ρ_y1y3_, ρ_y1y3|y2_, ρ_y1y3|y4_, and ρ_y1y3|y2y4_) are not significantly different from 0, arrowheads pointing to the common adjacent trait (y2) are added (y1 → y2 ← y3 in the graph of Step 2 in Figure 1). This kind of ‘V’ structure is called an ‘unshielded collider.’ The same process is repeated for the remaining possible set of traits.

### 2.3 Step 3

In the partially oriented graph obtained from step 2, as many undirected edges as possible are oriented without creating a new unshielded collider or cycle. In the case of the example in Figure 1, y2 and y4 are connected by an undirected edge. If an arrowhead pointing to y2 from y4 is added to the undirected edge (y2 ← y4), new unshielded colliders are created (y1 → y2 ← y4 and y3 → y2 ← y4); therefore, a new directed edge from y4 to y2 cannot be added. In contrast, arrowheads pointing to y4 from y2 can be added to the undirected edge (y2 → y4) because no new unshielded colliders are created. Finally, the graph of the causal structure is completed, shown as the graph in Step 3 of Figure 1. Additionally, an example of a cyclic graph is as follows: y1 → y2 → y3 → y1.

Statistical decisions regarding declaring partial correlations as null or not null are based on highest posterior density (HPD) intervals. If the interval contained the value 0, the correlation is declared as null. Outputs may differ according to the probability content used for the decisions.

In the context of the mixed‐model analysis, after fitting the MTM to the traits of interest, the IC algorithm can be applied to samples from the posterior distribution of the residual (co)variance matrix obtained from the multiple‐trait analysis because the residual (co)variances are considered to be information from the joint distributions of the traits conditional on genetic effects, which corrects the search for confounding because of such effects when they are correlated (Valente et al., 2010). The program ICPS written in R (R Development Core Team, 2009), which can carry out the IC algorithm analysis, is provided by Valente and Rosa (2013).

## 3 STRUCTURAL EQUATION MODEL

In the case of existing causal relationships between traits, as mentioned in the previous section, fitting a SEM (Haavelmo, 1943; Wright, 1921) can infer the causal coefficients between the traits. A SEM with a causal structure and random additive genetic effects, which was first proposed by Gianola and Solensen (2004), has been reviewed (Rosa et al., 2011; Wu, Heringstad, & Gianola, 2010) and the model with t traits can be written as follows:

$$y_{i}=\Lambda y_{i}+X_{i}\beta +u_{i}+e_{i}$$

where $y_{i}$ is a t×1 vector of phenotypic records on animal i; **Λ** is a t×t matrix of structural coefficients, which is filled by 0 except for the off‐diagonal elements that correspond to the causal structure; β is a vector of fixed effects; $u_{i}$ is a vector of random additive genetic effects; $e_{i}$ is a vector of random residual effects; and $X_{i}$ is a known incidence matrix. The joint distribution of vectors $u_{i}$ and $e_{i}$ in the equation is as follows:

$$\left[\begin{array}{c} u_{i}\\ e_{i} \end{array}\right]∼N\left\{\left[\begin{array}{c} 0\\ 0 \end{array}\right],\left[\begin{array}{cc} G_{0} & 0\\ 0 & \Psi_{0} \end{array}\right]\right\}$$

where $G_{0}$ is the additive genetic (co)variance matrix and $\Psi_{0}$ is the diagonal residual variance matrix.

**FIGURE 2:**
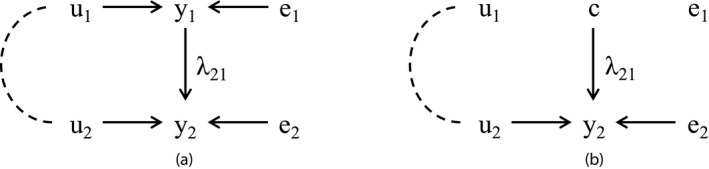
Hypothetical recursive causal structure involving two traits under no external intervention (a) and under external intervention (b). y_1_ and y_2_ are phenotypes; u_1_ and u_2_ are the additive genetic effects, which affect directly on y_1_ and y_2_, respectively, but u_1_ is also the indirect genetic effect on y_2_; e_1_ and e_2_ are the residual effects; λ_21_ is the causal coefficient; and c is a constant. Dashed arc represents genetic correlation between the additive genetic effects

By reducing the above SEM for y, the model is transformed, as indicated below (Gianola & Solensen, 2004; Varona et al., 2007):

$$\begin{array}{c} \left(I-\Lambda \right)y_{i}=X_{i}\beta +u_{i}+e_{i}\\ \;\;y_{i}={\left(I-\Lambda \right)}^{-1}X_{i}\beta +{\left(I-\Lambda \right)}^{-1}u_{i}+{\left(I-\Lambda \right)}^{-1}e_{i}\\ \;\;=\mu_{i}^{*}+u_{i}^{*}+e_{i}^{*} \end{array}$$

where $\mu_{i}^{*}={\left(I-\Lambda \right)}^{-1}X_{i}\beta ,\;u_{i}^{*}={\left(I-\Lambda \right)}^{-1}u_{i}$, and $e_{i}^{*}={\left(I-\Lambda \right)}^{-1}e_{i}$. In addition, the joint distribution of $u_{i}^{*}$ and $e_{i}^{*}$ is as follows:

$$\left[\begin{array}{c} u_{i}^{*}\\ e_{i}^{*} \end{array}\right]∼N\left\{\left[\begin{array}{c} 0\\ 0 \end{array}\right],\left[\begin{array}{cc} G_{0}^{*} & 0\\ 0 & R_{0}^{*} \end{array}\right]\right\}$$

with $G_{0}^{*}={\left(I-\Lambda \right)}^{-1}G_{0}\left(I-\Lambda \right)\prime^{-1},\;R_{0}^{*}={\left(I-\Lambda \right)}^{-1}\psi_{0}\left(I-\Lambda \right)\prime^{-1}$. Here, $u_{i}^{*}$, $e_{i}^{*}$, $G_{0}^{*}$, and $R_{0}^{*}$ are the similar vectors as described above ($G_{0}^{*}$ is the additive genetic (co)variance matrix and $R_{0}^{*}$ is the residual (co)variance matrix). Like this, the SEM can be transformed into a MTM, which ignores causal relationships between traits. Therefore, the likelihood between MTM and SEM is equivalent (Varona et al., 2007). However, genetic effects estimated from the two models have different meaning. The MTM only infers ‘overall’ genetic effects, which include all ‘direct’ and ‘indirect’ genetic effects on each trait, as illustrated later. Here, the indirect genetic effects are those that are mediated by other phenotype traits and the direct genetic effects are those that are not. In contrast, the SEM can infer the direct genetic effects, which are separated from the causal effects (corresponding to the indirect genetic effects; Valente, Rosa, Gianola, Wu, & Weigel, 2013). For instance, assuming the causal structure between two traits shown in Figure 2a, the SEM can be represented as follows:

$$y_{1}=u_{1}+e_{1}$$

$$y_{2}=\lambda_{21}y_{1}+u_{2}+e_{2}$$

where $y_{1}$ and $y_{2}$ are the phenotypes; $u_{1}$ and $u_{2}$ are the additive genetic effects which affect directly on $y_{1}$ and $y_{2}$, respectively (direct genetic effects), but $u_{1}$ is also the indirect genetic effect on $y_{2}$; $e_{1}$ and $e_{2}$ are the residual effects; and $\lambda_{21}$ is the causal effect from $y_{1}$ to $y_{2}$. In addition, the equation described above can be transformed as follows:

$$y_{1}=u_{1}+e_{1}=u_{1}^{*}+e_{1}^{*}$$

$$y_{2}=\lambda_{21}\left(u_{1}+e_{1}\right)+u_{2}+e_{2}=\left(\lambda_{21}u_{1}+u_{2}\right)+\left(\lambda_{21}e_{1}+e_{2}\right)=u_{2}^{*}+e_{2}^{*}$$

where $u_{1}^{*}$ and $u_{2}^{*}$, $e_{1}^{*}$ and $e_{2}^{*}$ are the additive genetic and residual effects in the MTM, respectively. This equation shows that the genetic effects in the SEM affect not only the corresponding phenotypes directly but also the other phenotypes indirectly via the causal structure. In addition, it suggests that the genetic effects in the MTM represent the overall effects, which also include the direct and indirect effects via the causal effects ($u_{2}^{*}=\lambda_{21}u_{1}+u_{2}$).

## 4 INTERVENTIONS IN THE SEM

As shown in the previous section, the SEM can infer direct and indirect genetic effects separately but the MTM cannot. Therefore, a breeding strategy based only on estimated genetic parameters obtained from the MTM would cause a delay in achieving the breeding goal if causal relationships and external ‘interventions,’ which would block indirect genetic effects, exist among the traits (Valente et al., 2013). An example of the intervention for $y_{1}$ in Figure 2a is shown in Figure 2b. The value of $y_{1}$ is fixed. The equation, which represents the intervention shown in Figure 2b, is as follows:

$$y_{1}=c$$

$$y_{2}=\lambda_{21}c+u_{2}+e_{2}$$

where c is a constant and the indirect genetic effect of $u_{1}\;\text{to}\;y_{2}$ via the causal structure no longer exists. Examples of the external intervention in management practices in livestock production systems are cross‐fostering for litter size in sows and cesarean sections for CD.

Lopez de Maturana, Wu, Gianola, Weigel, and Rosa (2009) inferred the causal effects of GL, CD, and stillbirth (SB) by fitting a mixed‐effects threshold SEM to the causal structure, assuming that GL affects CD and SB, and CD affects SB. Their results showed that the causal effects of GL to CD, GL to SB, and CD to SB were 0.47, 0.23, and 0.60, respectively. Using this result as an example, Valente et al. (2013) illustrated numerically that the artificial interventions in CD, such as cesarean sections in cows, would invert the ranking of genetic effects on SB because the indirect genetic effects of GL via CD and direct genetic effects of CD would be blocked in the causal structure. Therefore, in any breeding and production system based on multiple traits, it is important to study the potential causal relationships among the traits (Rosa et al., 2011).

If the causal effects can be inferred by fitting the SEM to the causal structure, giving an artificial intervention, such as management practice, to the variation in causing traits can phenotypically improve the following traits. In this way, the application of causal inference and fitting the SEM are effective not only for breeding programs but also for planning for management practices in livestock production systems (Rosa & Valente, 2013). Therefore, when causal relationships exist between target traits, application of the SEM to the traits should be considered.

## 5 APPLYING THE IC ALGORITHM AND SEM FOR ANIMAL BREEDING

Ever since Gianola and Solensen (2004) proposed adaptations of the SEM in the context of quantitative genetics and mixed‐effect models, several studies have applied the SEM to different species and traits in the context of animal breeding. de los Campos, Gianola, Boettcher, et al. (2006) and de los Campos, Gianola, and Heringstad (2006) studied the relationship between somatic cell score (SCS) and milk yield (MY) in dairy cows and dairy goats. This work is the first application of the SEM to field data. Similarly, in dairy cattle, there are many reports exploring the relationships between SCS or clinical mastitis and MY in Norwegian Red cows (Wu et al., 2007, 2008); between claw disorders and MY in Holstein cows (Konig et al., 2008); between health and fertility traits in Norwegian Red cows (Heringstad et al., 2009); among GL, CD, and SB in US Holstein cows (Lopez de Maturana et al., 2009, 2010); and between SCS and MY in Canadian Holstein cows (Jamrozik, Bohmanova, & Schaeffer, 2010; Jamrozik & Schaeffer, 2010). Some of the authors proposed extensions for the SEM, such as accounting for population heterogeneity (Wu et al., 2007), the Gaussian‐threshold Bayesian hierarchical model (Wu et al., 2008), the heterogeneous causal model (Wu et al., 2010), and the random regression model (Jamrozik et al., 2010; Jamrozik & Schaeffer, 2010). In swine, Varona et al. (2007) used a recursive model to analyze the relationship between litter size and average litter weight in Landrace and Yorkshire pigs and found likelihood equivalence between MTM and the SEM. Ibanez‐Escriche et al. (2010) developed a change‐point recursive model using change‐point detection techniques (Carlin, Gelfand, & Smith, 1992; Chib, 1998) and fitted the model to the litter size and number of SBs in Large White pigs. However, these studies assumed that causal structures were known a priori.

Recently, some studies fitted SEMs based on a data‐driven causal structure search using the IC algorithm. The IC algorithm fitted to the posterior distribution of residual (co)variance matrices that are considered phenotypic correlations conditional on additive genetic correlations between traits; this approach was proposed by Valente et al. (2010). They used simulated data and showed that this approach recovered the correct causal network. Then, Valente, Rosa, Silva, Teixeira, and Torres (2011) presented the first application of such a methodology to a real dataset by exploring the space of causal structures among five productive and reproductive traits in European quail. From the application of the IC algorithm with different HPD intervals, three undirected graphs were obtained, but all had no directed edge or unshielded collider. To construct the structures among the five traits and fit SEMs to the structures, the authors oriented the edges of the graphs according to temporal information as a prior knowledge.

Bouwman, Valente, Janss, Bovenhuis, and Rosa (2014) explored causal networks between 14 bovine milk fatty acids, which are highly correlated traits, by applying the IC algorithm. They retrieved undirected graphs using the IC algorithm with different HPD intervals, and then oriented the undirected graph obtained with the 95% HPD interval according to prior biological knowledge of de novo synthesis to fit the SEM: C4:0 → C6:0 → C8:0 → C10:0 → C12:0. The authors showed that inferred structural coefficients ranged from 0.85 to 1.05 and the SEM could be more plausible than the MTM by the deviance information criterion (DIC; Spiegelhalter, Best, Carlin, & van der Linde, 2002). In the genetic parameters obtained from the SEM, the genetic variances for downstream traits in the causal structure showed a gradual decrease (i.e., 0.460 for C4:0, 0.114 for C6:0, 0.073 for C8:0, 0.066 for C10:0, and 0.004 for C12:0), suggesting that indirect genetic effects from upstream traits were gradually explaining a larger portion of genetic variability.

In Japanese Black cattle, Inoue et al. (2016) inferred phenotypic causal structures and causal effects among meat quality traits. They applied the IC algorithm with different HPD intervals to meat quality traits, including beef marling score (BMS), beef color score (BCS), firmness of beef (FIR), texture of beef (TEX), beef fat color score (BFS), and the ratio of monounsaturated fatty acids to saturated fatty acids (MUS). They obtained a partially oriented graph except for BFS by using the IC algorithm with 80% of HPD intervals. The obtained graph had an undirected edge between FIR and TEX; therefore, two competing causal structures (i.e., DAGs), with either the arrow FIR → TEX or FIR ← TEX, were fitted using a SEM to infer structural coefficients between the selected traits. The authors finally suggested that the DAG with the directed edge of FIR ← TEX was more feasible for the structures based on the results of DICs of the SEMs. The structural coefficient for the path from MUS and BCS to BMS showed that a 1‐unit improvement in MUS or BCS resulted in an increase of 0.85 or a decrease of 0.54 in BMS in the DAG. They also reported that the genetic variances in the downstream traits (BMS, FIR, and TEX) in the causal structure from the SEM were smaller than those obtained from the MTM, whereas the variances in the upstream traits (BCS and MUS), which were not conditional on any of the other traits in the causal structure, had no significant differences between the SEM and MTM. This indicated that a breeding strategy based only on inferred genetic parameters obtained from the MTM would cause a delay in achieving the breeding goal if external interventions occurred in the upstream traits.

Inoue et al. (2017) also inferred causal structures and the causal effects among reproductive traits (CD and GL) and calf size (CS: birth weight, withers height, and chest girth of calves) in Japanese Black cattle. The IC algorithm with different HPD intervals was applied to the posterior distribution of the residual (co)variance matrix obtained from a threshold multiple‐trait sire–maternal grand sire (MGS) model. The results of the IC algorithm with 95% and 90% of HPD intervals only retrieved an undirected graph, which had edges between GL and CS and between CS and CD (i.e., GL – CS − CD). Then, they fitted the SEM to the causal structure that was assigned directions on the edges detected by the IC algorithm according to prior biological knowledge and graph theory (i.e., GL → CS → CD). The structural coefficients of GL on CD via CS on the observable scale showed that an extra day of GL increased the risk of dystocia by 0.2% (via chest girth) and 0.3% (via birth weight and withers height), in the causal structure. The authors also suggested that the application of the IC algorithm to the residual variance components from the sire–MGS model could lead to the detection of an incorrect structure because maternal genetic effects might not be removed completely from the residual variance components in the model.

More recently, techniques for inferring causal relationships by using the IC algorithm and SEM have applied to genome‐wide association studies (GWAS). Momen et al. (2018) applied SEM to GWAS on chickens, including causal relationships among breast meat (BM), body weight (BW), and hen‐house production (HHP), to compare the results obtained from both an SEM‐based GWAS (SEM‐GWAS) and a traditional multitrait association analysis (MTM‐GWAS). They set a causal structure, BM → BW and HHP and BW → HHP, found by the IC algorithm and fitted the SEM to the structure. As a result, the application of SEM‐GWAS could separate SNP effects into direct, indirect, and total effects. The authors concluded that the SEM‐GWAS delivered a more comprehensive understanding of SNP effects than did the MTM‐GWAS.

## 6 CONCLUSIONS

The results of the papers that are introduced in this review showed that the application of the IC algorithm to infer a causal structure is one of the most helpful tools for detecting an underlying causal structure without proper prior knowledge or with uncertain relationships between traits. The reports also indicated that visualization of the causal structure among traits on a graph and fitting the SEM to the causal structure could infer the effects of interventions, which are not given by correlations. Hence, information from the SEM provides more insights into and suggestions on breeding strategies than does that from a MTM, which is a conventional model usually used for multiple‐trait analysis. These new procedures could be utilized in the field of animal breeding.
